# What Defines an Age-Friendly Health System? Comment on "Developing a Conceptual Framework for an Age-Friendly Health System: A Scoping Review"

**DOI:** 10.34172/ijhpm.9374

**Published:** 2025-11-29

**Authors:** Margaret Wallhagen

**Affiliations:** Department of Physiological Nursing, School of Nursing, University of California, San Francisco, CA, USA.

**Keywords:** Health Systems, Life-Span, Healthy Aging, Conceptual Frameworks, Age-Friendly Health Systems

## Abstract

To address some of the changing needs of the increasing number of older adults, theories and conceptual frameworks designed to make environments and healthcare settings more welcoming to, and supportive of, older adults, have been proposed. Most recently, significant attention has been given to the concept of an "age-friendly health system." While the concept of age-friendly environments is not new, how such environments are operationalized varies, few data are available on outcomes, and there remains debate about the essential nature of key components of an age-friendly environment. This commentary discusses the conceptual framework proposed by Karami and colleagues published in the 2023 edition of the *International Journal of Health Policy and Management*. Questions are raised about the meaning of age-friendly and how it can be integrated into a life-span perspective of health.

 Aging is a dynamic process that evolves across decades and is manifest in multiple ways in any given individual and any given time in a life trajectory. However, there is generally an overall decline across the life-span in one’s ability to adapt to the health and environmental demands encountered, with older adults experiencing physiological vulnerabilities even while benefitting from life-long learnings on how to best face any given challenge. There is also an increase in the incidence of chronic illnesses which challenge adaptive capacities. To address some of the changing needs of older adults, theories and conceptual frameworks designed to make environments and healthcare settings more welcoming to, and supportive of, older adults, have been proposed. Most recently, significant attention has been given to the concept of an “age-friendly health system.”

 While the concept of age-friendly environments is not new, how such environments are operationalized varies and there is, of yet, few data on outcomes. In light of this, Karami and colleagues^1^ developed a very broad and inclusive conceptual framework designed to capture the key health system components necessary to support an age-friendly health system. The framework situates an age-friendly approach to the care of older adults within the broad socio-economic context, is inclusive of healthcare settings and emphasizes system processes or availability of resources rather than individual level care domains (Figure).

**Figure:**
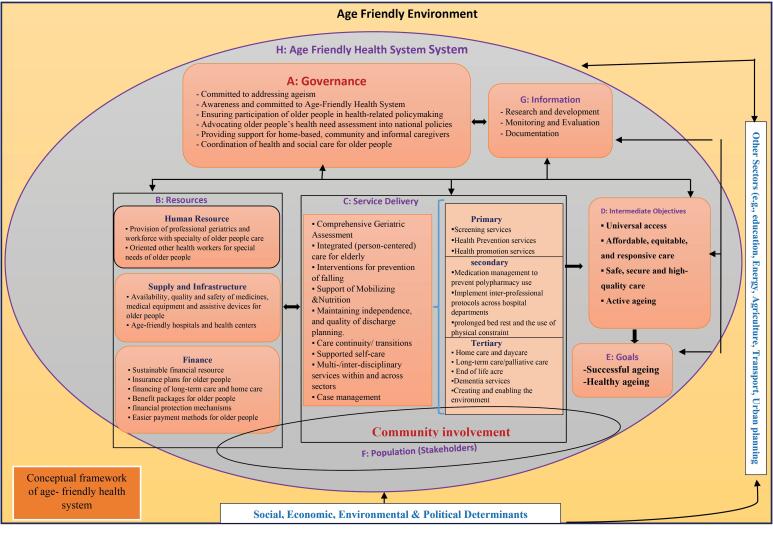
Karami and Colleagues’ Conceptual Framework of the Age-Friendly Health System. Reproduced with permission from Karami et al^1^ under the Creative Commons CC BY 4.0 license.

 In reviewing this conceptual framework and appraising its applicability, it is helpful to place the age-friendly movement in context. Thus, the purpose of this brief commentary is to reflect on several previous models designed to address the unique needs of older adults and then discuss the proposed conceptual framework in terms of its applicability as well as the questions it raises for future research.

 As early as the 1970s, Lawton and Nahemow^2^ presented the concept of “person-environment” fit whereby an individual’s ability to maintain well-being depended on the “fit” or match between the person’s functional capacity and resources and the demands imposed on the situation in which they lived. This was early in the environmental gerontology movement^3^ and was an important milestone in acknowledging the critical role the physical as well as social environment played in the health and well-being of older adults. Additionally, the concept of “fit” is relevant to how individuals experience health conditions as these impose specific demands that challenge the individual’s adaptative, internal capacities as well as external, environmental resources, such as living situation and family support

 Concurrently, other models of care evolved designed to support the health and well-being of older adults or others with chronic conditions. These include the Chronic Care Model (CCM)^4^ and Comprehensive Geriatric Assessment (CGA).^5^ These models or approaches to care, like the concepts expressed within the environmental gerontology movement, emphasized the importance of matching the needs and wishes of older adults with the care provided while also recognizing the intra-individual heterogeneity that becomes increasingly common across the life-span. Specifically, the CCM was designed to improve care by recognizing that chronic care took place within three overlapping contexts, community, health system, and provider organization, and by using a patient-centered approach.^3,6^ While noted to be broadly adopted^4^ it has not impacted broad system change and does not address the broader needs of older adults such as health promotion and chronic illness prevention.

 Unlike the CCM, the CGA was designed to improve the care of older adults by focusing on more than a specific disease. Rather, as Ferrucci and Orini^5^ noted, “health, functional status, and quality of life of older persons cannot be summarized by the sum of diseases but they are rather affected by behavioral, social, environmental, financial, and political factors” (p. 1-2). The domains of CGA are broad and include assessment of not only medical conditions but also cognitive, functional, affective and economic status; environmental issues; social support; and spirituality.^7^ Given the breath of the domains, a CGA is aligned with the data needed to provide patient-centered and age-friendly care. At the same time, a range of barriers to actualizing the goals of CGA have been identified^8,9^ including inadequate buy-in, lack of staff training, and communication issues as well as time. Further, disparities in care processes and outcomes for older adults continue, suggesting the need for new or more comprehensive models that are more fully integrated into the health system broadly.

 This is one of the goals of the age-friendly health system, specifically as articulated within the United States. The John A. Harford Foundation (JAHF), in collaboration with the Institute for Healthcare Improvement and in partnership with the American Hospital Association and the Catholic Health Association of the United States collaborated in the delineation of what was essential to an “age-friendly” health system. As noted by the JAHF, “An age-friendly health system would keep healthy older adults healthy, be proactive in addressing potential health needs, prevent avoidable harms, improve care of those with serious illness and at the end of life, and support family caregivers throughout” (p. 23).^10^ They decerned the key elements as defined by the “4Ms”: What Matters, Mentation, Mobility, and Medication. This focus is very much patient centered as the central construct is “What Matters” and all else really flows from assuring the care supports the person’s ability to achieve or maintain their goals and values.

 While this specific approach is still relatively new, the number of associations and groups involved in its development and the long-standing influence of the JAHF on the care of older adults provides a platform from which to build a broader consensus. Along these lines, the Centers for Medicare and Medicaid recently released an Age Friendly Hospital measure.^11^ All hospitals participating in the Centers for Medicare and Medicaid Hospital Inpatient Quality Reporting Program are now required to report on how they are complying with these measures. The measures closely align with the 4Ms: Eliciting Patient Healthcare Goals; Responsible Medication Management; Frailty Screening and Intervention; Social Vulnerability; and Age-Friendly Care Leadership. If the hospital is unable to state compliance, they could possibly face significant financial penalties. This is, thus, a new level of incentive to begin to increase incorporation of age-friendly principles into at least the in-patient setting. Although this is possibly an important step, it is specific to the health system as operationalized within the United States and there remain specific issues to overcome. Data suggest, as with other models, operationalizing the age-friendly approach faces barriers^12^ including adequate staff training, buy-in, and person-family engagement.

 From this abbreviated historical lens, it is possible to reflect back on the comprehensive conceptual model proposed by Karami et al which takes a global and societal perspective. Specifically, the conceptual framework suggests that all components of society are active players, including broad governmental active engagement and support in diminishing ageism and assuring that older adults are active participants in decision making around policy. The framework includes the need for a workforce well-trained in geriatrics and with the necessary resources to meet the determined needs. Given the breath of the framework, it is hard to argue that it does not address the multiple types of services and resources that could support care for older adults and, as such, it could serve as a heuristic that could be used to identify key areas that need further research and/or policy discussion to assess best practices. Karmi et al should also be lauded for their attempt to capture the broad domains of an age-friendly health system. Further, several other recent papers have also attempted to refine our understanding of an age-friendly health system and provide some support for components of Karmi and colleagues’ conceptual model. A systematic review that also evolved a conceptual framework of an age-friendly health system and that was similarly published in 2024 identified many of the same areas of focus.^13^ The difference in the latter is that it included a more person focused approach and identified key attributes of age-friendly care: Respect for older adults autonomy and needs; Leadership and organizational knowledge and support; Proactive policies and processes of care; Holistic care environment; and Communication and follow-up. Additionally, a concept analysis recently accomplished by Fan and colleagues^14^ identified similar attributes of an age-friendly health system: adapting to the developmental needs, promoting the autonomy and engagement, and a sense of ease and burden-free for older adults. The value of the latter is that its analysis included articles from non-western countries, thus possibly supporting the broad applicability of the basic concepts of an age-friendly health system.

 However, while the underlying concepts may be broadly applicable, how these are operationalized may need to be adapted to various health systems and cultures. For example, in their discussion of the Age-Friendly Cities initiative in Taiwan, Chao and Haung^15^ argue that there needs to be an oriental paradigm based on specific cultural differences with the western paradigm: individualism versus collectivism, universalism versus particularism, and low power distance versus high power distance. Operationally, they suggest this might entail taking collectivism into consideration during a needs assessment, acknowledging that the community leader’s perspective may be important in planning, and using a more top-down than bottom-up approach to implementation.

 Additionally, while possibly functioning as a heuristic, the Karami et al framework and the growing number of articles trying to refine the concept of age-friendly raise several issues that warrant further exploration and research – issues that are not inherently unique to the age-friendly movement. The framework currently does not provide guidance on how these various sectors interact, communicate, and coordinate, both within and across domains. This is one of the major impediments to coordinated care currently experienced by the multiple levels of health systems, from acute to chronic to community-based to long term care. More data are needed on the types of information that are critical to assure coordination across care settings. With the advanced technologies now available, strategies to capture essential health and care elements should be possible that can move with the person within and across settings. A broader understanding of and support for the age-friendly initiative may also benefit from what Coyle and colleagues^16^ call a “spillover” effect whereby there is diffusion of age-friendly processes across systems. While the latter is focused on age-friendly cities, achieving the type of integration suggested by Karmi et al necessitates this cross-sector adoption. To promote such integration, there needs to be more education of healthcare providers and community workers on the care of older adults and the meaning of “age-friendly.”^17^

 The Karmi et al framework also suggests this is an approach for all older adults yet experience from implementation of the CGA suggests that targeting services is the most cost efficient and effective way to maximize positive outcomes. The question is, who benefits most from an “age-friendly” environment and which elements are especially critical? In the United States, most Acute Care for the Elderly services that are designed to meet the needs of acutely ill older adults in hospital settings set age limits on whom they provide services for. In light of resource scarcity, more data are needed in the types of settings that benefit most from a full range of age-friendly approaches. What are the key essential aspects of an age-friendly setting? Is it emphasizing the “what matters” to the individual? And can we capture the evolution in what matters across time with awareness of how individual’s views on end-of-life care often evolve. This emphasizes the need for appropriate metrics to assess the actual impact of age-friendly systems. Few data are available to-date on outcomes.

 We may also need to ask, what truly defines “age-friendly”? The various models, in general, come from a very western perspective that values individualism and autonomy. Can age-friendly environments be developed that incorporate the multiple cultural and health values of a range of persons and backgrounds as suggested by Chao and Haung?^15^ Additionally, can older adults articulate “what matters” to them? Many of us live our values yet cannot articulate what they are. Can we support this? Much of the literature emphasizes the value of persons “being in control” and yet not everyone obtains a sense of control by “being in control.”^18^

 Finally, how can this framework articulate with other frameworks focusing population health across the life-span? Along these lines, it may be valuable to look to the new World Health Organization’s (WHO’s) Framework to Implement a Life Course Approach in Practice.^19^ While life-span perspectives are not new, the value of the WHO framework is the broad engagement in its development and its acknowledgement that aging begins at birth and that maintaining health across the life-span provides a strong base for maintain health as we age. The framework also emphasizes the value and importance of cross generation communication and engagement. In reality, the WHO and age-friendly frameworks are not mutually exclusive. Rather, it would be valuable to explore best practices to integrate an age-friendly environment during the latter phases of a life trajectory, identify key metrics to assess outcomes and refine interventions, and assure that there is not disparity in resource allocation. From this perspective, the Karami et al conceptual framework can serve as a resource that identified key system domains needed to support age-friendly care.

## Disclosure of artificial intelligence (AI) use

 Not applicable.

## Ethical issues

 Not applicable.

## Conflicts of interest

 Author declares that she has no conflicts of interest.
